# Immune Checkpoint Targets for Host-Directed Therapy to Prevent and Treat Leishmaniasis

**DOI:** 10.3389/fimmu.2017.01492

**Published:** 2017-11-08

**Authors:** Rajiv Kumar, Shashi Bhushan Chauhan, Susanna S. Ng, Shyam Sundar, Christian R. Engwerda

**Affiliations:** ^1^Department of Biochemistry, Institute of Science, Banaras Hindu University, Varanasi, India; ^2^Department of Medicine, Institute of Medical Sciences, Banaras Hindu University, Varanasi, India; ^3^QIMR Berghofer Medical Research Institute, Brisbane, QLD, Australia

**Keywords:** leishmaniasis, immunoregulation, immunotherapy, pathology, cell-mediated immunity

## Abstract

Leishmaniasis encompasses a group of diseases caused by protozoan parasites belonging to the genus *Leishmania*. These diseases range from life threatening visceral forms to self-healing cutaneous lesions, and each disease manifestations can progress to complications involving dissemination of parasites to skin or mucosal tissue. A feature of leishmaniasis is the key role host immune responses play in disease outcome. T cells are critical for controlling parasite growth. However, they can also contribute to disease onset and progression. For example, potent regulatory T cell responses can develop that suppress antiparasitic immunity. Alternatively, hyperactivated CD4^+^ or CD8^+^ T cells can be generated that cause damage to host tissues. There is no licensed human vaccine and drug treatment options are often limited and problematic. Hence, there is an urgent need for new strategies to improve the efficacy of current vaccine candidates and/or enhance both antiparasitic drug effectiveness and subsequent immunity in treated individuals. Here, we describe our current understanding about host immune responses contributing to disease protection and progression in the various forms of leishmaniasis. We also discuss how this knowledge may be used to develop new strategies for host-directed immune therapy to prevent or treat leishmaniasis. Given the major advances made in immune therapy in the cancer and autoimmune fields in recent years, there are significant opportunities to ride on the back of these successes in the infectious disease domain. Conversely, the rapid progress in our understanding about host immune responses during leishmaniasis is also providing opportunities to develop novel immunotherapy strategies that could have broad applications in diseases characterized by inflammation or immune dysfunction.

## Leishmaniasis

Leishmaniasis describes a collection of neglected tropical diseases caused by protozoan parasites of the genus *Leishmania* that are transmitted by female *Phlebotomine* sand flies (1). It largely affects the poorest populations living in developing countries and is prevalent throughout the tropical and subtropical regions of Africa, Asia, the Mediterranean, Southern Europe, and South and Central America. Globally, 350 million people are at risk of developing leishmaniasis and 1.5–2 million new cases occur annually (2). The clinical spectrum of leishmaniasis ranges from the life-threatening visceral form to self-healing cutaneous lesions or a more serious mucosal manifestation.

Visceral leishmaniasis (VL), also known as kala-azar, typically involves long-term, low-grade fever, enlarged spleen and liver, weight loss, pancytopenia, and hypergammaglobulinemia [reviewed in Ref. (3)]. Untreated VL cases are almost always fatal, and more than 90% of cases occur in India, Bangladesh, Nepal, Sudan, Ethiopia, and Brazil with an estimated incidence of at least 500,000 new cases and 50,000 deaths each year (2–4). Of note, the state of Bihar in north east India has been the focus of most VL cases for many years (5), but recent efforts toward elimination, and civil unrest in Southern Sudan, have now made the latter region the source of most cases (6). Post kala-azar dermal leishmaniasis (PKDL), which can be a late cutaneous manifestation of VL, either following drug treatment or sometimes independent of VL development, is confined to the Indian subcontinent [India, Nepal and Bangladesh, and East Africa (Sudan)]. It presents as an accumulation of heavily infected macrophages in the skin (7–9), which appear as nodules, papules, or hypopigmented macules. PKDL can appear from 6 months to years after apparent VL cure in the Indian subcontinent, but can also occur earlier (within 6 months) or along with VL in the Sudan, where the incidence of this disease is higher. PKDL heals spontaneously in a proportion of cases in Africa, but rarely in Indian VL patients and requires prolonged treatment. Since PKDL patients harbor increased parasite numbers in their skin, they are thought to act as parasite reservoir and play an important role in disease transmission in endemic regions. As such, they may be an important population to target with effective host-directed immunotherapies.

Cutaneous leishmaniasis (CL) is characterized by the development of ulcerative skin lesions containing parasites and is the most common form of disease occurring mainly in Afghanistan, Algeria, Brazil, Colombia, the Islamic Republic of Iran, Pakistan, Peru, Saudi Arabia, and Syria (2, 10). Cutaneous lesions are generally localized and may persist for months to years or heal spontaneously within weeks. The development of disfiguring scars at the affected skin areas following healing is a major concern.

Mucocutaneous leishmaniasis (MCL) is prevalent in Bolivia, Brazil, Peru, and Ethiopia (WHO, 2014) and is caused by *L. baziliensis, L. panamensis*, and *L. aethiopica*. These species metastasize to mucosal tissue in the mouth and upper respiratory tract, leading to localized tissue destruction. MCL can present from several months to years after the development of a cutaneous lesion. Diffused cutaneous leishmaniasis (DCL), which is more common in central and South America, is thought to occur in immunosuppressed individuals, where parasites can readily disseminate to subcutaneous tissue. As both MCL and DCL are associated with strong and weak host inflammatory responses, respectively, which appear to contribute to disease pathology, they also have potential for improved treatment involving host-directed immune therapy.

## Immunological Characteristics of Disease

### Visceral Leishmaniasis

Studies in experimental VL in mice, caused by infection with the human parasites *L. donovani* or *L. infantum*, show the development of antiparasitic IFNγ-producing, Tbet^+^ CD4^+^ T (Th1) cells is critical for resistance against infection (11). Many VL patients fail to generate potent cell-mediated immune responses against parasite antigens and this is thought to be an underlying cause of disease. However, enhanced IFNγ mRNA expression in the spleen and bone marrow, as well as increased circulatory plasma IFNγ, TNF and IL-12 in VL patients, suggests that they do not lack a protective Th1 response, but instead, immunosuppressive mechanism are established to prevent parasite killing (12–15). Importantly, antigen-specific responses in whole blood assays indicate that VL patient CD4^+^ T cells have the capacity to produce IFNγ in response to parasite antigen (16–18). Therefore, attention has now focused on regulatory mechanisms that prevent Th1 cell-mediated control of parasite growth.

CD4^+^ T cell IL-10 production has emerged as an important mechanism to dampen T cell activation in parasitic infections, including in humans with VL (15, 19, 20). Importantly, most T cell-derived IL-10 is not produced by thymus-derived Foxp3-expressing regulatory T (Treg) cells. Instead, the IL-10 producing CD4^+^ T cells often co-produce IFNγ and have been designated type 1 regulatory (Tr1) cells (15). They are increasingly recognized as a critical regulatory CD4^+^ T cell subset that protects tissue from inflammation (21–23). However, Tr1 cells also appear to promote infection by suppressing Th1 cell-mediated immunity. The role of IL-10 in immune suppression and disease progression is well documented in both experimental and human VL (15, 24–28). Human VL is associated with enhanced IL-10 plasma levels, increased IL-10 mRNA expression in lymph nodes, bone marrow, and spleen, and readily detected IL-10 produced by whole blood cells from VL patients following parasite antigen stimulation (15, 28). IL-10 dampens major histocompatibility complex (MHC) class II expression on APC and downregulates TNF and nitric oxide (NO) production, leading to reduced parasite clearance and suppressed activation of Th1 cells (29). Neutralization of IL-10 in VL patient sera can suppress *L. donovani* replication in macrophages (15, 30), and IL-10 blockade in splenic aspirate cultures from VL patients can limit parasite replication and enhance Th1 cell cytokine production (24). IL-10 can also modulate immune responses by promoting T cell exhaustion (31, 32).

A number of immune checkpoint molecules have also been identified on CD4^+^ T cells from experimental VL models and VL patients (Table 1). These include CTLA-4 (CD152) and PD-1, which are negative regulators of T cells and are expressed on exhausted or anergic T cells during chronic infection. CTLA-4 binds to the costimulatory ligands B7-1 (CD80) and B7-2 (CD86), with much higher affinity than CD28, while PD-1 interacts with PD-1 ligand 1 (PD-L1; B7.H1) and PD-L2. Activation of CTLA-4 leads to increased levels of TGFβ, as well as apoptosis of CD4^+^ T cells in murine VL (33). In mice infected with *L. donovani*, CTLA-4 blockade decreased parasite burden in both liver and spleen, associated with increased frequencies of IFNγ and IL-4 producing cells, and an accelerated hepatic granulomatous response (34, 35). CTLA-4 blockade has also been shown to increase the efficiency of chemotherapy in *L. donovani* infected mice (36, 37). Similarly, blockade of PD-1 or PD-L1 resulted in enhanced parasite clearance and increased pro-inflammatory cytokine production in experimental VL (38–40). Thus, these studies clearly show the therapeutic potential of targeting immune checkpoint molecules for host-directed immune therapy in VL.

**Table 1:** Immune checkpoint molecules tested for therapeutic effects in leishmaniasis.

	Disease				Biological system		Reference
	Visceral leishmaniasis	Cutaneous leishmaniasis	Diffused cutaneous leishmaniasis	Mucocutaneous leishmaniasis	Human	Preclinical	
IL-10	Y	Y			Y	Y	(15, 24, 26, 27, 41–45)
PD-1	Y	Y	Y		Y	Y	(38, 40, 46–50)
PDL-1/2		Y					(49)
CTLA-4	Y				Y	Y	(34–37, 51, 52)
OX40	Y	Y				Y	(37, 53, 54)
CD40	Y	Y				Y	(36, 55–57)
CD28	Y					Y	(36)
CD80/86		Y				Y	(58–61)
ICOS		Y				Y	(62)

*Y, indicates immune checkpoint molecule tested in the disease indicated*.

Another important regulatory cytokine involved in VL is IL-27, which is composed of the EBI-3 and p28 sub-units. IL-27 belongs to IL-6/12 cytokine family and was originally described as a co-factor for Th1 cell differentiation, along with IL-12 (63, 64). IL-27 promotes T cell IL-10 production in mice, which is further amplified by autocrine IL-21 production (65, 66). IL-27 receptor-deficient mice infected with *L. donovani* developed enhanced Th1 responses, but this was associated with severe liver pathology (67). Patients with active VL also presented with enhanced IL-27 plasma levels, as well as increased mRNA transcripts encoding EBI-3 and p28 in splenic aspirates (28). IL-27 produced by CD14^+^ cells, along with IL-21 from T cell sources, promoted the differentiation and expansion of Ag-specific, IL-10–producing T cells in VL patients. Importantly, pro-inflammatory cytokines, such as IFNγ, act on macrophages and stimulate IL-27 production, suggesting a feedback mechanism to stimulate IL-10 production to control IFNγ levels and protect host tissue. IL-27 has also been associated with suppression of CD4^+^ T cell IL-17A and IL-22 secretion (68, 69). Since *L. donovani* antigen-stimulated production of both IL-17A and IL-22 by PBMC in an apparent disease-resistant Sudanese population, these cytokines were proposed to be protective following *L. donovani* infection and, therefore, elevated IL-27 in VL patients might not only promote disease by increasing IL-10 production, but also by regulating IL-17 production (70). Studies with Indian VL patients showed low levels of IL-17A mRNA transcripts, as well as the IL-17-related transcription factor RORγT during active disease (28). However, there was no direct evidence that this Th17 response was suppressed by IL-27. Furthermore, there is evidence from experimental VL that the impact of IL-17A may depend of the stage of infection, whereby this cytokine impedes antiparasitic immunity early (71), but is protective following establishment of infection (72). Hence, immune dysfunction in VL patients appears to involve multiple immune regulatory pathways that differ both spatially and temporally, and identifying which can be safely and effectively targeted for clinical advantage should be a major research priority.

Other immunosuppressive mechanisms established during VL may be mediated through regulatory T cells. Regulatory T cells can be classified as thymus-derived CD4^+^CD25^+^FoxP3^+^ T (Treg) cells and inducible regulatory T cells that include conventional T cells that convert to FoxP3^+^ regulatory T cells in peripheral tissues, as well as Tr1 cells (73, 74). To maintain immune homeostatic conditions, Treg cells limit the activity of potentially self-reactive T cell responses and prevent immune-mediated pathology and autoimmunity (75, 76). However, these same mechanisms may also contribute to impaired pathogen clearance during parasitic infection. Treg cells function by secreting regulatory cytokines such IL-10 and TGFβ, as well as expressing inhibitory molecules such as CTLA-4 and IL-35 (77). Treg cells express high levels of CD25 (IL-2R), thereby allowing them to form the high affinity receptor for IL-2, which allows them to deprive conventional T cells of this important growth factor, thus causing apoptosis (78). However, there is little evidence for the involvement of Treg cells in human or experimental VL. Studies from VL patients in Bihar, India showed no accumulation of Treg cells in the spleen or blood, and the frequency of these cells did not change during the course of infection (15, 79). Moreover, FoxP3^−^ T cells were the major source of IL-10 mRNA in VL patient spleens, and this finding was in accordance with murine VL studies where IL-10 secretion by FoxP3^−^CD4^+^ T cells correlated with disease severity (19). However, other studies have reported the accumulation of Treg cells at sites of infection and suggested their possible role in disease pathogenesis in both human and experimental VL. One study from India suggested that Treg cells were a major source of IL-10 in the bone marrow of VL patients and that IL-10 secretion from Treg cells suppressed conventional T cells (80). In a different study with Indian VL patients, production of IL-10 and TGFβ by Treg cells was positively correlated with parasite load (81). Similarly, TGFβ-producing Treg cells were shown to accumulate in infected tissues in a murine model of VL (82). Thus, further investigation is needed to establish whether Treg cells are involved in the pathogenesis of VL and whether they can be modulated for therapeutic advantage. Interestingly, TGFβ is also secreted by macrophages and dendritic cells (DCs) during experimental VL. A cathepsin B-like cysteine protease present in *L. donovani* can activate TGFβ (83), which, in turn, activates arginase-1, leading to enhanced l-ornithine production and reduced NO secretion, thereby promoting parasite survival in infected cells (84, 85). Human VL patients have enhanced TGFβ plasma levels (13) during active disease, suggesting a possible role in pathogenesis. However, more research is needed to better understanding the precise mechanisms of TGFβ-mediated suppression of antiparasitic immunity before it can be considered as an immune therapy target.

**Figure 1:**
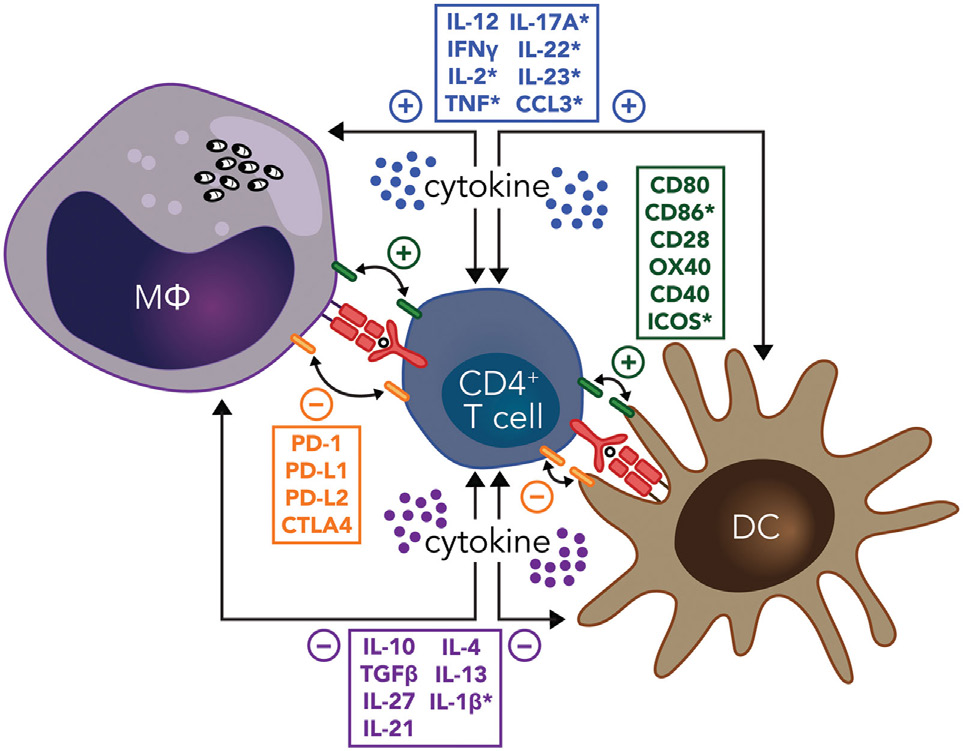
Potential immunotherapy targets to manipulate CD4^+^ T cell-dendritic cell (DC) and CD4^+^ T cell-infected macrophage (MΦ) interactions during leishmaniasis include both cognate and soluble cytokine signals. Primary signals between CD4^+^ T cells and macrophages or DCs through major histocompatibility complex class II antigen presentation of parasite peptide to the T cell receptor are indicated (red), as are both positive (green), and negative (orange) costimulatory signals. Positive (blue) and negative (purple) soluble cytokine signals are also show. Note that many of these signals are bidirectional, as indicated by the double-ended arrows. In addition, molecules highlighted by an asterisk have been reported to have the opposite effects in different type of *Leishmania* species infections. Amastigotes residing in macrophages are shown in black.

The development of regulatory DC subsets following *L. donovani* infection can also have a major impact on T cell responses during VL. These regulatory DCs are capable of producing anti-inflammatory cytokines, such as IL-10, TGFβ, and IL-27 (86). In experimental VL, it was shown that IL-10^+^IL-27^+^ DCs were able to promote IL-10 production by Th1 cells *in vivo* and identified this cell population as a potential target for immunotherapy (87). Furthermore, CD11c^lo^CD45RB^+^ DCs in the spleen of *L. donovani*-infected mice had high levels of IL-10 production, compared to CD11c^hi^ populations, and displayed features of immature DCs, including low expression of co-stimulatory molecules and intracellular MHC class II (88). These DCs also produced IL-10 when stimulated with lipopolysaccharide and promoted Treg cell IL-10 production capable of inhibiting mixed lymphocyte reactions driven by conventional DCs (89). The inhibitory effects of these regulatory DCs could be reversed by IL-10 signaling blockade, indicating that IL-10 production was a critical regulatory mechanisms employed by this DC subset (88). Therefore, both cognate and soluble cytokine signals between effector CD4^+^ T cells, DCs, and infected macrophages have key roles in determining whether parasite growth is controlled and/or disease develops, making these interactions promising targets for immune therapy (Figure 1).

CD8^+^ T cells can kill *Leishmania*-infected macrophages by secreting cytolytic enzymes (90, 91). However, studies with human VL blood samples suggest that CD8^+^ T cells have an anergic or exhausted phenotype, as indicated by high expression of IL-10, CTLA-4, and PD-1, which may hamper the protective efficiency of these cells during active disease (51). A better understanding of role of CD8^+^ T cells during VL is needed if the antiparasitic potential of these cells through vaccination or immune therapy can be exploited.

## Post Kala-Azar Dermal Leishmaniasis

Post kala-azar dermal leishmaniasis often develops as a cutaneous complication of VL in apparently cured patients, but can also develop independent of VL. The pathogenesis of PKDL remains poorly understood. It has been postulated that immune suppression may allow multiplication of latent parasites from the viscera or residing in the skin (8). Similar to clinical VL, elevated IFNγ and TNF levels are found in lesions of PKDL patients, with the concurrent presence of the immunosuppressive cytokines IL-10 and TGFβ (92). Despite the presence of high IFN-γ and TNF in these tissues, there is reduced expression of IFNγ and TNF receptors in Indian PKDL patients (92, 93), while genetic polymorphisms in the IFNγ receptor 1 gene promoter region have been reported and found to be associated with susceptibility to PKDL in Sudanese patients (94, 95). Treg cells have also been associated with PKDL in the Indian subcontinent and elevated FoxP3, CD25, and CTLA-4 mRNA expression has been reported in the skin of patients. Furthermore, Foxp3, CD25, and IL-10 mRNA levels directly correlated with parasite load in these PKDL patients (96). Since PKDL either develops soon after VL or independent of VL in the Sudanese population, but takes longer to develop after VL in the Indian subcontinent, the immunopathology of PKDL is likely to differ in these populations. PKDL patients from the Sudan display immune responses similar to cured VL patients and their PBMC proliferate in response to parasite antigens and CD4^+^ T cells secrete IFNγ and IL-10 (97, 98). However, PKDL patients from the Indian subcontinent have high numbers of CD8^+^ T cells in their lesions and circulation, along with increased antigen-induced IL-10 production by circulating CD8^+^ T cells and impaired antigen-induced proliferation (99, 100). Studies with Indian PKDL patients have also demonstrated enhanced Th17 cell responses by analyzing mRNA and protein expression of Th17-related IL-23, IL-17A, and RORγt (101). Stimulation of PKDL patient PBMCs with parasite antigens resulted in IL-17A and IL-23 production, while stimulation with recombinant IL-17A enhanced TNF and NO production. Hence, these data suggest that enhanced Th17 responses may have a role in parasite clearance during PKDL. However, it is still not clear whether regulatory cytokines and/or other mechanisms suppress IL-17-mediated protective responses during active disease. This knowledge is important if we wish to manipulate this immunoregulatory pathway to improve antiparasitic immunity.

## Cutaneous Leishmaniasis

Cutaneous leishmaniasis is caused by several *Leishmania* species, including *Leishmania major, L. braziliensis, L. mexicana, and L. amazonensis*. Cell-mediated immune responses at the site of cutaneous lesions are of primary importance in determining the outcome of disease. Furthermore, in murine models of CL caused by *L. major*, the genetic background of mice also determines disease outcome. In C57BL/6 mice, Th1 cell responses promote a self-healing process, while Th2 responses are associated with parasite persistence in the lesions of BALB/c mice [reviewed in Ref. (102, 103)]. In humans infected with *L. major*, cutaneous lesions have been associated with high IFN-γ, IL-10, and IL-12 mRNA accumulation, indicative of a mixed CD4^+^ T cell response. Several immune checkpoint molecules have also been identified in experimental CL studies that can modify CD4^+^ T cell responses to favor parasite clearance (Table 1), again demonstrating immune checkpoint blockade as a potential approach to improve disease treatments.

Following transmission of *L. major* to mice *via* sand fly bites or needle, neutrophils rapidly infiltrate the bite site and capture injected parasites (104). Neutrophils rapidly express apoptotic markers following *L. major* uptake, which attracts monocytes and DCs to the site of infection for removal of apoptotic cells (105, 106). This allows the uptake of parasites into phagocytic cells without triggering inflammation, and thereby enabling establishment of infection. Infected neutrophils also express chemokines, such as CCL-3, to attract DCs to the site of infection (107). This may help to stimulate Th1 cell responses following activation of DCs through interaction of DC-SIGN on DC and specific glycans on neutrophils (108). In addition, CCL3 can induce IL-12 secretion by macrophages in C57BL/6 mice, but not in BALB/c mice (107). Given these latter pro-inflammatory properties of neutrophils, any modifications of neutrophil functions may have to be directed specifically toward their activity as a “Trojan horse” for establishment of infection. However, it is important to remember that the role of neutrophils is critically dependent on the *Leishmania* species in question, the parasite lifecycle stage and stage of infection, when trying to manipulate neutrophil functions.

Following *L. major* infection, complement-dependent platelet activation, including the release of platelet-derived growth factors, can stimulate the release of CCL2/MCP-1 by leukocytes and mesenchymal cells, leading to recruitment of Ly6C^+^ inflammatory monocytes, which can capture and kill parasites *via* oxidative burst (109). Importantly, these monocytes can migrate to lymph nodes and differentiate into specialized DC subsets during *L. major* infection. These monocytes-derived DCs secret high levels of IL-12 and stimulate *L. major*-specific Th1 cell responses, suggesting a contribution to protection against disease (110, 111). Monocytes expressing high levels of CCR2 can also capture *L. major* at the site of infection in C57BL/6 mice, then migrate to draining lymph nodes and differentiate into inducible nitric oxide (iNOS)-producing DC that also promote Th1 cell-mediated protection (112). Hence, the promotion of these activities in the context of vaccination or drug treatment may be desirable.

NK cells are also recruited to the site of infection in mice infected with *L. major* and produce IFN-γ, which can amplify DC IL-12 production required for the development of strong Th1 cell responses (113). However, NK cells can also produce IL-10 during *L. donovani* infection (114), suggesting they can play either antiparasitic or immunoregulatory roles during infection. Depending upon the dose of infection, CD8^+^ T cells also produce IFN-γ in murine models of CL, which can also help shape early adaptive immune responses associated with protection (115–118). However, resolution of infection following *L. major* infection is primarily associated with CD4^+^ T cell-mediated immunity (119–121). Despite healing of cutaneous lesions, parasites continue to persist at the original site of infection, in part due to IL-10-mediated mechanisms, and these persisting parasites are thought to help maintain effector memory CD4^+^ T cells (T_EM_) that protect against re-infection (122, 123). This T_EM_ response is lost if parasites are eliminated, as shown by studies in which mice were manipulated to achieve sterile cure (122). Thus, concomitant immunity is compromised and protection against a secondary challenge can be lost in the absence of persisting parasites (124). However, there is also evidence that a pool of long-lasting central memory CD4^+^ T cells (T_CM_) can develop in absence of persisting parasites, and that these can acquire effector functions after re-infection leading to protection (125). These T_CM_ cells require additional IL-12 signals to develop into fully functional Th1 cells, and in absence of this signal, they can convert into IL-4-producing Th2 cells (126). T_CM_ cells appear to be generated early during infection, and not only help in controlling secondary infections, but also contribute to clearance of primary infection (127). Hence, these findings suggest both T_EM_ and T_CM_ cells participate in maintaining immunity to *L. major* infection, but only the T_EM_ require persistent parasite antigen. Therefore, vaccines designed to protect against leishmaniasis should target the expansion of long-lasting T_CM_ cells, rather than short lived T_EM_ cells.

More recently, skin resident memory (T_RM_) CD8^+^ T cells have been shown to provide protection against *L. major* infection, independent of circulatory CD4^+^ T cells, by recruiting inflammatory monocytes, which rapidly control parasite growth *via* reactive oxygen species (ROS) and NO generation (128). Thus, these T_RM_ cells also represent a potential target cell population for vaccination. Treg cells appear to play a role in *L. major* persistence in C57BL/6 mice by suppressing CD4^+^ T cell effector functions through IL-10-mediated immunosuppressive mechanisms (122). The IL-10 produced by these Treg cells can also promote parasite persistence by modulating APC function and/or inhibiting parasite killing mechanisms in infected macrophages. Thus, the activity of Treg cells at the site of infection can promote concomitant immunity, but also allow parasites to persist. Therefore, although Treg cells could be targeted for immunomodulation, care would have to be taken to ensure that long-term protection was not compromised.

In non-healing CL caused by *L. major* Seidman strain in C57BL/6 mice, Nlrp3 inflammasome-dependent IL-1β activation plays an important role in determining disease outcome (129). The activation of the Nlrp3 inflammasome enhanced IL-1β activation through caspase-1 cleavage, which caused recruitment of neutrophils to the site of infection, and ultimately resulted in the suppression of immunity, which was confirmed by using neutropenic Genista mice (129). Nlrp3 can promote Th2 cell development in non-healing cutaneous lesions caused by *L. major* infection in BALB/c mice (130). Thus, this inflammasome and related cell signaling pathways are potential targets for immune therapy to treat and promote healing of cutaneous lesions in human CL (see also below). However, inflammasome- and caspase-1-dependent IL-1-β production has been shown to provide resistance against *L. amazonensis* infection in mice by triggering NO production (131), thus emphasizing the need for careful consideration in choosing appropriate targets for immune modulation in specific disease settings.

## Mucocutaneous Leishmaniasis

Although self-cure is often the outcome of CL, some patients infected with *L. braziliensis, L. panamensis*, and *L. aethiopica* can develop MCL after resolution of their primary lesion, characterized by chronic inflammation of the nasal mucosa and by a hyperactive T-cell response (132, 133), associated with high levels of pro-inflammatory cytokines, such as IFN-γ and TNF-α, and decreased levels of IL-10 and TGF-β (132, 134, 135). Thus, a poorly regulated T cell response is an underlying cause of disease pathogenesis in MCL patients. In patients infected with *L*. *braziliensis*, the number of CD8^+^ T cells recruited to lesions increased as disease progressed, and these cells expressed high levels of granzymes and perforin, indicating they had elevated cytolytic activity (136). In fact, these CD8^+^ T cells have now been shown to contribute to inflammation and disease pathology *via* perforin-mediated cytotoxicity (137). In mice co-infected with lymphocytic choriomeningitis virus and *L. braziliensis*, it was found that perforin-mediated CD8^+^ T cell cytotoxicity in the lesion resulted in enhanced recruitment of neutrophils and monocytes, which produced IL-1-β that contributed to immunopathology and disease severity (138). Importantly, pharmacological blockade of Nlrp3 reduced inflammation caused by cytotoxic CD8^+^ T cells in this mouse model of MCL, thus identifying this inflammasome, as well as CD8^+^ T cell-mediated cytotoxicity, as potential targets for immunotherapy. This was supported by additional data from the same study, using skin biopsies and PBMCs from CL patients infected with *L. braziliensis*, which IL-1β was highly expressed in skin lesions and blockade of the Nlrp3 inflammasome prevented the IL-1-β secretion from skin biopsies, suggesting a similar pathogenic mechanism might be operating during clinical MCL.

In addition to CD8^+^ T cell-mediated pathology, Th17 cells have also been associated with pathogenesis in MCL patients (139). MCL lesions were found to have elevated IL-17A mRNA, as well as TGF-β, ROR-γT, and IL-23 mRNA levels, which are associated with Th17 cell differentiation. Interestingly, IL-17 was not only produced by CD4^+^ T cells but also by CD8^+^ T cells, CD14^+^, and CCR6^+^ Cells. The enhanced production of IL-17 was associated with infiltration and recruitment of neutrophils into the lesion, suggesting that IL-17 may promote inflammatory responses in MCL patients. Thus, IL-17 production could be a therapeutic target in MCL patients to reduce tissue pathology.

## Diffuse Cutaneous Leishmaniasis

Diffused cutaneous leishmaniasis is a severe manifestation of CL characterized by a defective cellular immune response to *Leishmania* antigens (140). However, this unresponsiveness is restricted to antiparasitic immune responses, as responses to unrelated antigens remain intact (141, 142). DCL patients have high parasite numbers within skin lesions, which has been associated with low levels of IFN-γ and IL-2 mRNA, and concurrent high levels of IL-10, IL-4, and IL-5 mRNA in lesions (135). Therapeutic cure was associated with enhanced IFNγ production, but low IL-10 expression (143), indicating the requirement for a classical Th1 cells response for favorable clinical outcomes. This disease is also associated with high antibody titers and plasma TGF-β [reviewed in Ref. (102)]. IL-10 and TGF-β, along with Treg cells, can antagonize IFN-γ and TNF activities, resulting in impaired microbicidal activities in infected macrophages [reviewed in Ref. (144)]. However, it is not clear in DCL whether high antigen exposure causes T cell unresponsiveness or if impaired T cell responses promote localized parasite growth in the skin [reviewed in Ref. (144)].

Diffused cutaneous leishmaniasis patients respond poorly to conventional drug treatment (145), but some degree of treatment success has been achieved with immune modulation using IFN-γ combined with viable BCG and antimonial drug (146, 147). Since unresponsive T cells often express inhibitory molecules, such as PD-1, CTLA-4, and LAG-3, these may make attractive targets for immune therapy in DCL patients. IL-1-β has also been associated with disease severity in *L. mexicana*-infected DCL patients (148), making it another potential therapeutic target. Again, care will need to be taken to ensure the promotion of antiparasitic immunity in this context is not at the expense of protection against tissue damage.

## Leishmanization

The fact that *L. major*-induced cutaneous lesions often heal spontaneously and protect against future infection is the basis for leishmanization, which involves inoculation with live, virulent parasites in an unexposed part of body to produce a controlled lesion. This strategy has been practiced successfully in the former Soviet Union, Middle East, and Israel, and likely provides protection in humans because it mimics a natural infection, including allowing parasite persistence and development of concomitant immunity. The protection provided by leishmanization is essentially T cell-mediated, whereby IFN-γ-producing CD4^+^ T cells are recruited to dermal sites of infection where they perform effector functions, including the promotion of microbicidal mechanisms in infected macrophages (123). Importantly, the success of leishmanization depends on the viability and infectivity of injected parasite. Parasites that lost virulence stimulated delayed-type hypersensitive reactions, but did not provide protection from natural re-infection (149). Leishmanization was abandoned in most countries because of logistical problems and safety concerns, due to some immunosuppressed individuals developing non-healing lesions (150). Interestingly, leishmanization can provide cross protection against the visceral form of disease, as leishmanized C57BL/6 mice infected with *L. major* were protected from heterologous visceral infection with *L. infantum*, associated with recruitment of IFN-γ-producing Ly6C^+^CD4^+^ T cells to both skin and visceral organs (151). Similarly, longitudinal studies in the Sudan indicated that people residing in an *L. major* endemic area were protected against VL caused by *L. donovani* (152, 153). In addition, CL caused by a *L. donovani* strain in Sri-Lanka provided cross protection against visceral disease (154). These findings suggest that leishmanization could be a strategy employed to increase protection against VL. However, a better understanding of the immunoregulatory mechanisms associated with this process is needed to fully exploit the positive aspects of leishmanization with improved safety.

## Strategies to Improve Vaccines

Although different *Leishmania* species cause a broad range of clinical symptoms, genetic analysis indicates a large degree of genomic conservation between species. Thus, it may be possible to generate broadly effective vaccines against different clinical diseases. However, despite many efforts, there is no effective, licensed vaccine to prevent human leishmaniasis. There is a major need for more efficacious and less toxic adjuvants and immune therapies for better vaccines for patients suffering from leishmaniasis. Studies in VL patients and experimental models (15, 155) indicate the rapid development of immunoregulatory networks following exposure to parasites, which raises questions about how these regulatory networks might influence subsequent immunity, particularly to vaccines. It is noteworthy that many vaccines tested in disease endemic regions have not performed as well as when tested in healthy volunteers. For example, the RTS,S/AS01 vaccine in children and infants affords 36 and 25% efficacy against clinical malaria, respectively (156), while a recent study showed that the efficacy of the same vaccine in healthy volunteers in CHMI studies was 52% (157). Similarly, BCG-mediated protection against pulmonary tuberculosis varies geographically and appears to be much less effective in areas with high incidence of previous infection with *M. tuberculosis* or sensitization with environmental *Mycobacteria* (158, 159). Although many reasons could account for the reduced efficacy of vaccines in disease endemic areas, these results suggest that the early establishment of potent, pathogen-specific immunoregulatory networks may be an important factor contributing to this problem (160). Treatment in the field of cancer has been revolutionized by immune checkpoint blockade strategies. These take advantage of the patients own immune system to recognize and kill cancer cells. Although many of the molecules being targeted by this approach were discovered in infectious diseases research, this approach has not been applied to reducing the burden of infection. Therefore, incorporating inhibitors of specific immune checkpoints into vaccine formulations may be one way to transiently reduce immune suppression to allow the generation of robust vaccine-mediated, antiparasitic immunity.

Both CTLA-4 and PD-1 blockade have been successfully used individually and in combination to treat cancer patients (161). Given that leishmaniasis is a chronic infection and shares several key immunoregulatory features with cancer, one strategy could be to “piggy back” on the success of immune checkpoint blockade drugs in cancer to either improve drug treatment protocols by making subsequent immunity more potent and long-lasting or enhance vaccine efficacy. However, it will be important to bear in mind that specific types and combinations of immune checkpoint blockade work best for particular types of cancer, and this is also likely to be the case with the spectrum of diseases caused by *Leishmania* species. Thus, careful consideration will need to be given to types of immune checkpoint blockade best suited to VL, CL, MCL, PKDL, or DCL because they are likely to differ in their outcomes.

## Strategies to Improve Drug Treatment

Antimonial chemotherapy was the mainstay for VL treatment for many decades (162). However, parasite resistance against these drugs has developed, especially in the Indian subcontinent (163, 164). Therefore, these drugs are now mainly employed to treat VL in Africa, while drugs such as Amphotericin B, Ambisome, Miltefosine, and paromomycin have been introduced to treat VL in areas of antimonial drug resistance (164, 165). However, these drugs are not without problems, such as toxicity, high cost, potential development of parasite drug resistance, and prolonged treatment regimes [reviewed in Ref. (165)]. Recently, a single dose of Ambisome (lipid formulation of Amphotericin B) was found to be sufficient to successfully treat VL with low toxicity and has now been recommended as a choice of treatment in India subcontinent (166, 167). The oral drug miltefosin has also been used in combination with Amphotericin B. However, based on studies in preclinical models of leishmaniasis, there are concerns that even with combination therapy, drug resistance will develop (168). Further, these drugs do not cause sterile cure and parasites persist in the infected individuals after drug treatment (168–170). This is concerning because these persisting parasites may help promote transmission, with people living in the same household being most at risk of infection (171, 172). Thus, not only should successful cure of disease be a goal of treatment, but lowering the burden of persisting parasites as far as possible is also desired if parasite transmission is to be minimized. However, when considering these goals, it will be important to remember that persistent parasites are also required to maintain concomitant immunity (170), so sterile cure of infected individuals may not necessarily be beneficial. Instead, it may be necessary to establish the level of parasite burden that is low enough to prevent parasite transmission, but at the same time, maintain concomitant immunity, and then try and achieve this through a combination of antiparasitic drug and immunomodulatory strategies.

Drug treatment works most effectively in association with the host immune system, and in particular, cell-mediated immune responses (164). Hence, understanding immunological changes during the course of infection and how these might be modulated to work best with drug is important. The use of biological molecules to stimulate cell-mediated immunity to help achieve therapeutic success has been tested in both preclinical and clinical studies on leishmaniasis (173). In an experimental model of leishmaniasis, treatment with recombinant IL-12 or anti-IL-10 receptor monoclonal antibody, along with pentavalent antimony (Sb^v^), resulted in improved clearance of *L. donovani* parasites, compared with animals treated with drug alone (27, 174). In addition, human recombinant IFN-γ has been successfully used to accelerate antiparasitic and clinical responses when used with antimony treatment, and importantly, treat seriously ill VL patients with refractory disease (175–178). Many drugs used to treat VL not only kill parasites, but also promote host immunity. For example, antimonial drugs stimulate the generation of ROS and NO, while miltefosine and Ambisome induce the secretion of IFN-γ, TNF, IL-12, IL-6, and IL-1β from immune cells with a simultaneous decrease in anti-inflammatory cytokine production (179–182). Thus, combining immune-based therapy with conventional antiparasitic drugs is an obvious strategy to improve current treatment protocols.

Rather than supplement or block immune effector molecules, another approach to use host-directed therapy to improve drug treatment is to target intracellular signal transduction pathways. For example, it has been shown that *L. infantum* infection rapidly induces activation of phosphatidylinositol 3-kinase/Akt and extracellular signal-regulated kinase1/2 in bone marrow-derived dendritic cells (BMDDC), thereby limiting their maturation and pro-inflammatory cytokine secretion. The blockade of this pathway with wortmannin resulted in reduced infection rates of BMDDC (183). Similarly, the rapid activation of protein tyrosine phosphatase, such as SHP-1, by *Leishmania* is another important parasite evasion strategy, and administration of the SHP-1 inhibitor bpV-phen to mice infected with *L. major* and *L. donovani* promoted control of infection *via* induction of reactive nitrogen intermediates that would otherwise be repressed by parasite-activated SHP-1 (184, 185). Hence, small molecule inhibitors of key cell signaling pathways is another potential approach that could be used with current antiparasitic drug treatment protocols.

## Developing Strategies for Resource Poor Setting

Leishmaniasis is generally a disease associated with poverty (186) and as such, diagnosis, treatment, and hospitalization costs are an important consideration in disease control programs. In addition, drug development programs for leishmaniasis are often not a high priority for pharmaceutical companies. Furthermore, even if effective, high-cost drugs or vaccines are available, they are unlikely to be used without significant government or philanthropic subsidization. Hence, a practical challenge is to supply relatively cheap drugs in resource poor settings, and this will require the participation of regulatory bodies, as well as public and private sector partnerships. A successful example of this was the implementation of the wider use of Ambisome for VL treatment. This is normally a high-cost drug, but has been substantially reduced in cost for the distribution through the public sector agencies in developing countries, by an agreement negotiated between WHO and the manufacturer (187). However, other issues can complicate such arrangements. For example, the requirement of a reliable cold chain for Ambisome implementation can result in a failure to provide the drug or the use of drug stored under conditions not consistent with storage advice. Therefore, the introduction of new treatment regimes for diseases such as VL will need to consider multiple aspects of drug development, formulation, storage, and delivery. One approach for reducing development costs is to repurpose drugs already licensed for other indications, as was the case with Ambisome, which was first licensed as an anti-fungal drug. Another way of reducing cost is to develop cheaper small molecules rather than more expensive biologics to target parasites or host responses. In addition, as well as usual safety considerations, the stability of drugs in areas of unreliable cold-chain must be considered, as well as ease of manufacturing. Therefore, when considering strategies to promote host directed therapy, regardless whether this be targeting specific immune check points or stimulating microbicidal mechanisms, small molecules are likely to be more cost-effective than antibodies.

## Concluding Remarks

Leishmaniasis has clear, unmet medical needs. These differ, depending on the disease in question. However, the host immune response to infection is a central component of each of these diseases, whether it is immune dysfunction in the case of VL and DCL or immune-mediated tissue pathology in the case of severe CL and MCL. Therefore, targeting these host responses, as is increasingly occurring in other chronic diseases, such as cancer and autoimmunity, offers promising new opportunities to either improve the efficacy of vaccine candidates or drug treatment protocols. Given the long time lines for vaccine development, the latter approach may have a greater impact in the short term. Furthermore, combining host-directed therapy with antiparasitic drug, offers the added advantage of further reducing parasite loads in treated individuals and improving long-term protective immunity. These outcomes will greatly benefit current disease elimination programs.

## Author Contributions

RK, SC, SN, SS, and CE wrote the paper. SN and SC prepared the figures and table.

## Conflict of Interest Statement

The authors declare that this research was conducted in the absence of any commercial or financial relationships that could be construed as a potential conflict of interest.
